# Perspective: Current Pitfalls in the Search for Future Treatments and Prevention of Parkinson's Disease

**DOI:** 10.3389/fneur.2020.00686

**Published:** 2020-07-08

**Authors:** Peter C. Poortvliet, Karen O'Maley, Peter A. Silburn, George D. Mellick

**Affiliations:** ^1^School of Environment and Science, Griffith Institute for Drug Discovery, Griffith University, Brisbane, QLD, Australia; ^2^School of Nursing, Midwifery and Social Work, University of Queensland, Brisbane, QLD, Australia; ^3^Queensland Brain Institute, University of Queensland, Brisbane, QLD, Australia

**Keywords:** Parkinson's disease, complex syndrome, pre-diagnostic period, biomarker, disease modification

## Abstract

We are gradually becoming aware that there is more to Parkinson's disease (PD) than meets the eye. Accumulating evidence has unveiled a disease complexity that has not (yet) been incorporated into ongoing efforts aimed at slowing, halting or reversing the course of PD, likely underlying their lack of success. There is a substantial latency between the actual onset of PD pathology and our ability to confirm diagnosis, during which accumulating structural and functional damage might be too advanced for effective modification or protection. Identification at the earliest stages of the disease course in the absence of Parkinsonism is crucial if we are to intervene when it matters most. Prognostic and therapeutic inferences can only be successful if we are able to accurately predict who is at risk for developing PD and if we can differentiate amongst the considerable clinicopathologic diversity. Biomarkers can greatly improve our identification and differentiation abilities if we are able to disentangle cause and effect.

## Parkinson's Disease-Modification and Neuroprotection are Not Yet Available

Despite efforts to develop new treatments that can slow, stop or even reverse the trajectory of PD (disease modification) and preserve neural integrity and function (neuroprotection), none have yet been successfully demonstrated (1). The primary reason for this lack of success remains our incomplete understanding of the exact cause(s) of PD, and factors involved in subsequent disease progression (2). Arguably, many of the previous clinical trials aimed at developing new treatments were methodologically and conceptually flawed (2) by assuming that PD can be defined as a single diagnostic entity, without taking into consideration the complexity, diversity and timing of pathogenesis (3–5). Furthermore, past study designs show little or no regard for the state of neuronal degeneration at time of enrollment, or the inter- and intra-individual clinicopathologic heterogeneity (2). This is exemplified by the PD models used to investigate potential new treatments, which have been criticized for their lack of complexity and true representation of the natural course of PD in humans (3). In human trials, the sensitivity and specificity of outcome measures have also received considerable scrutiny, as most are highly subjective, still firmly rooted in the motor domain and unable to accurately assess therapeutic target engagement (6). *In vitro* cellular modeling using person specific stem cells or induced pluripotent stem cells has shown considerable potential as a method to closely reproduce specific pathological circumstances and directly study neurodegenerative processes and mechanisms and the effects of interventions (7–9). In that regard, *in vitro* cellular modeling has been referred to as “the most robust and phenotypically similar model for PD” (8). The limitation of course is that the complexity of PD is still not fully accounted for, with the requirement to focus on specific aspects of PD while ignoring others (9). Complementary approaches that can mitigate for the unaccounted aspects would be required in order to advance.

We will fail to make progress in the development of new therapeutic strategies until we take into consideration the full natural history of the disease process and associated clinicopathologic diversity under this banner.

In the current perspective we aim to discuss emerging concepts and recent insights into the natural history of PD that will be important to consider before viable disease-modifying therapies can become a reality.

## PD is More Than Meets The Eye

**Figure 1:**
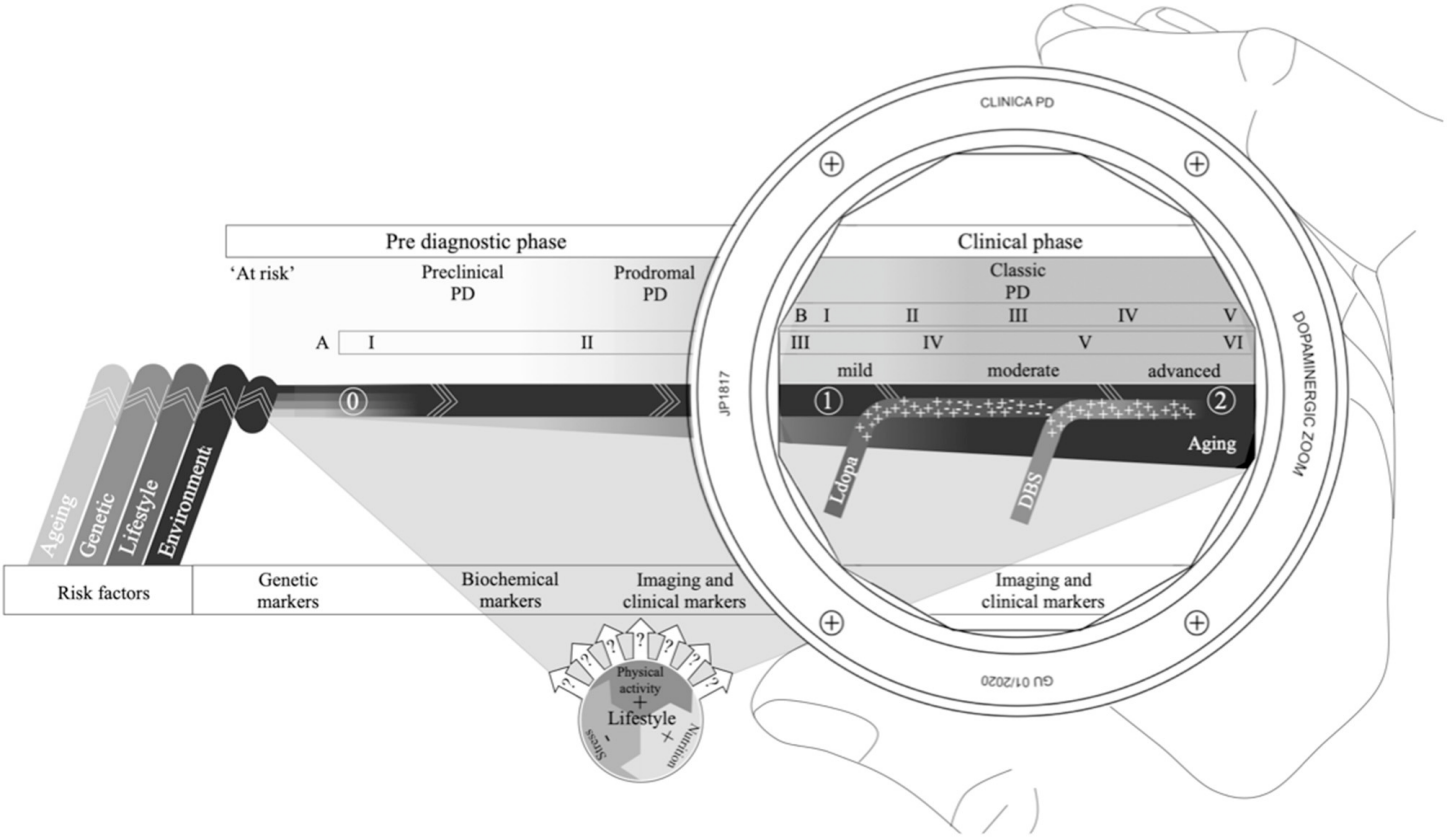
The PD continuum with an extended disease course that includes a combination of risk factors that can pre-dispose or cause PD (0); a preclinical phase, where the pathology has started but no signs are present; a prodromal phase, where non-motor symptoms dominate, but motor symptoms can be present; and the classic or clinical phase often viewed through a dopaminergic lens, delineated by the clinical diagnosis (1) and mortality (2), with Levodopa therapy and/or deep brain stimulation (DBS) forming essential therapies for symptomatic management. Several risk factors (like age and lifestyle) also have the potential to affect disease progression. The Braak six-point staging scheme (A) spans across the entire PD continuum, while the Hoehn & Yahr symptom progression scale, for reference (B), is confined to the classic PD phase. Different biomarkers are better suited at different points along the continuum depending on pathological and clinical evidence.

To date, clinical and scientific approaches to PD have mainly focused on few primary features, subsequently reducing it to a single diagnostic entity and viewing symptomatology through a dopaminergic lens (Figure 1) (10, 11). The characteristic clinical features in PD are observed as a deterioration of motor function expressed as bradykinesia, resting tremor, muscular rigidity and postural instability (12). The underlying pathological characteristics include an ongoing gradual loss of dopaminergic neurons in the nigrostriatal pathway, as well as the presence and proliferation of eosinophilic inclusions called Lewy bodies and Lewy neurites (11, 12). Over time, neural integrity becomes increasingly compromised eventually leading to an unsustainable dopamine deficiency ultimately resulting in functional complications and subsequent disability.

As definitive diagnostic confirmation is still only possible through *post-mortem* histopathological examination, available diagnostic criteria aim to increase the level of clinical diagnostic certainty *in vivo* (13). Current diagnostic criteria require the presence of a combination of cardinal motor symptoms to establish Parkinsonism, a group of neurological disorders with overlapping pathologic and symptomatic expressions (14). A combination of additional supportive features, red flags and exclusion criteria for differential diagnosis, then serve to further strengthen the clinical diagnostic certainty for PD, resulting in either clinically established or clinically probable PD (14). Symptomatic management, by way of compensation for the ensuing dopamine deficiency, remains the gold standard of clinical treatment (15). Although symptomatic management is successful at maintaining quality of life, especially during earlier stages of PD, long-term pharmacotherapy is associated with development of treatment-related motor complications that are difficult to manage (11). Furthermore, treatment options for non-motor symptoms remain limited (16).

Motor symptomatology is still considered the defining characteristic of PD; however, it is now widely recognized that a range of non-motor features (Table 1) form an integral part of the symptomatology (17). Although dopaminergic cell loss is considered the predominant pathological hallmark of PD, degeneration is not restricted to the nigrostriatal pathway. Neurotransmitter deficiency due to extranigral degeneration, including the serotonergic, noradrenergic, cholinergic, GABAergic, and glutamatergic systems, underlie numerous neuropsychiatric, autonomic, sensory, and sleep disorders, as well as the non-levodopa responsive motor symptoms of PD (18, 19). Furthermore, neuropathological evidence suggests that the presence of α-synuclein aggregates and Lewy pathology also extends to extranigral structures, including the cerebral cortex, olfactory structures, brainstem, spinal cord and even peripheral tissues (20). Naturally expressed throughout the CNS and many other tissues, α-synuclein is a presynaptic protein (21, 22). The exact function is still unknown, but α-synuclein is thought to play a role in the regulation of neurotransmitter release (23). Increased expression and accumulation of abnormal α-synuclein aggregates is thought to be neurotoxic and associated with pathological processes of PD (22, 23).

**Table 1:** An overview of the most common non-motor features by category.

**Category**	**Non-motor symptom**
Autonomic	Constipation^**^
	Salivation
	Bladder dysfunction
	Sexual dysfunction
	Respiratory dysfunction
	Cardiovascular dysfunction
	Fatigue
	Excessive sweating
Mood and behavior	Depression^**^
	Anxiety^**^
	Panic attacks
	Impulse control disorder
	Visual hallucinations^*^
	Delusions^*^
	Dementia
	Apathy
Sensory	Pain
	Olfactory dysfunction^**^
	Insomnia
Sleep	REM sleep behavior disorder^**^

* *Mostly medication related*.

** *Common prodromal symptoms*.

## Is it Time To Redefine PD?

Not only do these pathological findings provide an explanation for the wide range of non-motor symptomatology, indicating a more complex and systemic nature of PD, they also hint toward possible extranigral origins and earlier disease onset. To that effect, Braak et al. (20) proposed a six-point staging system, based on post-mortem histopathological evidence of abnormal α-synuclein accumulation throughout the nervous system of individuals with differing disease durations. They describe a rather systematic propagation of α-synuclein aggregates along interconnected neural networks, starting in the lower brainstem and anterior olfactory system and progressing to cortical areas with advancing disease. The pathology only reaches dopaminergic cells in the *substantia nigra* toward stages three and four, relating to the classic motor symptomatology.

In an effort to explain the extranigral origin, Braak et al. (24) proposed a dual hit hypothesis where an environmental pathogen likely enters the body through the nasal and gastric routes and then spreads via the dorsal motor nucleus of the vagus nerve and olfactory bulb to more central neuronal structures. A prion-like concept of disease progression has since been put forward that proposes a cell-to-cell spread of abnormal α-synuclein (25, 26) The concept was the result of findings of neuronal grafting studies, where, at autopsy after more than a decade of survival, host-to-graft propagation was found in some of the transplanted dopaminergic neurons (25). Since then several studies using animal and cellular models have supported the α-synuclein transmission concept (27–31). This is also problematic for new treatments efforts that focus on regeneration, where patient specific induced pluripotent stem cells are transplanted. Apart from the risks associated with regeneration medicine, such as unwanted biological effects and immune response, toxicity, neoplasm formation, disease transmission, reactivation of latent viruses, to rejection of the cells by the body (7), the transplanted cells would again be susceptible to α-synuclein propagation, compromising their long term health. Further evidence now suggests that different species or strains of α-synuclein can exert different effects depending on their folded state (32). Different oligomeric forms in particular seem to have different pathogenic effects including toxicity, which is suspected to contribute to the clinicopathological diversity of PD (33).

Although there is considerable support for the Braak hypothesis, some studies have shown that not all PD cases follow the systematic pathological progression (34). Higher stage pathology and subsequent symptomatology, such as primary dementia with Lewy bodies, has been found in absence of pathology in lower stage structures (35, 36). The presence of Lewy pathology in otherwise healthy individuals is also well-recognized (37). Furthermore, some genetic variants of PD do not express characteristic Lewy pathology. In addition, the considerable pathological and symptomatic heterogeneity of PD undermines strict systematic progression (20). Different patterns of pathological progression are most likely underlying the considerable clinical variability seen in PD. Studies have since shown that Lewy pathology and α-synuclein spread can occur in bi-directional manner along interconnected networks (38). This can partly explain some of the discrepancies in the Braak hypothesis, but considerable discussion remains on the topic.

As it stands, the traditional concept of PD as just a movement disorder is gradually making way for a more comprehensive and encompassing definition that recognizes the innate complexity of PD as a syndrome and the multiple affected neuroanatomical structures (nigral and extranigral) that lie at the foundation of the broad symptomatic range. Redefining PD as a multi-system neurodegenerative disorder (39) not only acknowledges the widespread spatial organization of neurodegeneration and possibly a peripheral origin, but also implies earlier temporal progression along a much more extended disease continuum.

## The Concept of PD Without Parkinsonism

It is now widely accepted that the classic PD course actually represents a relatively late stage of a broader process of disease (40). The extended PD course acknowledges a considerable pre-diagnostic phase, during which the underlying pathology has commenced, but symptomatology is either absent, non-specific or too subtle to meet current diagnostic criteria (1) (Figure 1). The pre-diagnostic phase is commonly further subdivided into an “at risk” phase, a preclinical or premotor phase and a prodromal phase, depending on clinicopathologic manifestations (40).

The earliest phase in the PD continuum, when the pathology is thought to have commenced, but clinical signs and symptomatology are lacking, is referred to as the preclinical phase (40). As the pathology progresses, compromises to neural integrity and function steadily increase to a point where symptomatology becomes manifest (41). During this prodromal phase, several non-motor symptoms are especially common (Table 1), including olfactory dysfunction, constipation, anxiety, depression, sympathetic denervation and REM sleep behavior disorder (40, 42). The non-motor features associated with the prodromal phase are non-specific and are generally easily disregarded as common aspects of normal aging (41). However, most, if not all individuals with PD have indicated the presence of one or more of these features prior to their diagnosis (42). Subtle motor symptoms also start to emerge during the prodromal phase as the underlying pathology slowly progresses (42). It is worthwhile noting that for clinical and scientific purposes subdividing the PD course, whether classic or pre-diagnostic, into different phases can be a meaningful way to deal with the complexity. In reality, definite phases are almost certainly unlikely and the PD course, in all probability, represents a continuum of transient states along which multiple factors continuously interact, with positive or negative impact (43).

If we are to move forward clinically and scientifically, we first need to come to grips that PD can be present in the absence of Parkinsonism. We then need objective and reliable measures to accurately identify those at risk of developing PD or those in the earliest developmental stages when traditional motor symptomatology has not (yet) emerged.

## Current Treatments are Too Little, Too Late To Affect Progression

As mentioned in the previous section, PD is now considered much more than just a movement disorder and the pathology extends well-beyond the nigrostriatal neural networks, potentially even originating in sites peripheral to the CNS. This has considerable implications on how we need to consider the timing of key milestones in the disease trajectory. It is now evident, that by the time the cardinal motor features manifest and diagnosis can be made, a vast majority of dopaminergic cells have already been lost (1, 44). The underlying pathology has been able to spread insidiously for years and compensatory mechanisms are no longer able to cope with the steadily increasing dopamine deficiency, resulting in overtly observable motor features (11, 45). In this regard, the cardinal motor features, traditionally used as diagnostic criteria, should instead be considered determinants of clinical progression of PD. Since most clinical trials are designed with PD diagnosis as minimum inclusion criterion, we argue that the compromises to neural integrity and function at this stage are already too advanced for disease modifying or protective therapies to take effect (1, 5). This stark realization is further supported by the fact that, on average, very few dopaminergic terminals remain in the striatum as early as 5 years following a formal clinical diagnosis and the commencement of dopaminergic therapy (46). At the moment, however, these motor symptoms are the only criteria available to guide PD diagnosis and subsequent therapeutic approaches. Any attempt at disease modification would have to commence as early as possible and this will require a reconsideration of how and when the diagnosis is made, what specific disease-related processes need to be targeted and how aggressive these need to be treated.

## How Can We Identify Those at Risk Before The Emergence of Symptoms?

The specific causes of PD remain unknown, and there is no clarity as to when the actual onset of PD occurs (47). Moreover, endophenotypes associated with early stages in the PD continuum are also factors that may pre-dispose for the development of classic movement PD (48) (Figure 1). Thus, there is a major challenge in distinguishing between true “symptoms” of a disease process from “risk factors” that are “associated” but neither necessary nor sufficient to result in disease.

Combinations and interactions of risk factors (e.g., lifestyle, environment, genetic, and aging) might differ between individuals, which may explain the considerable clinical and pathological diversity of PD. Although the risk factors can offer important clues for the pre-disposition of developing PD, even in the presence of certain risk factors, we currently lack the ability to accurately predict if and when pathological conversion will occur in most instances. As mentioned earlier, PD onset most likely does not involve a single triggering event, but is rather the consequence of a sequence of transient aggravating processes that tip the balance and sets the pathological progression in motion further along the disease continuum.

As the contribution of individual risk factors is thought to be relatively small, gene-environment interactions and how they can inform prediction of future PD in neurologically healthy populations have received considerable attention (49). Risk stratification studies, for instance, have started to model incidence scores using a range of known risk and prodromal factors and assigning each a value before calculating their predictive scores using specific algorithms or regression models (50–54). None of these models have yet been incorporated into clinical practice and have only been used for research purposes. Careful consideration of risk stratification attempts and many other investigations that try to elucidate the cause, progression and heterogeneity of PD reveals an ongoing difficulty in our ability to distinguish between cause and effect (55). Included factors are often based on observational associations, which lack essential definitive conclusions to make causal inferences and may be the result of inverse causation (55, 56). As eloquently pointed out by Chen (57), symptoms expressed in the pre-diagnostic phase several years before diagnosis, but at the time not suspected to be part of PD, might have impacted the factors that are now thought of as protective, such as smoking, physical activity, caffeine consumption. For instance, physical activity might be reduced in individuals in the prodromal stages of PD because of their prodromal features and probably not the other way around (57). It is important to realize that etiological factors may play different roles in the cause and/or progression of PD and would have to be monitored over long periods of time before we can make meaningful interpretations about their positive or negative implications.

## Can Biomarkers Help To Usefully Stratify Cases According To Causality?

The closer we get to the beginning of the PD continuum, the greater the reliance on pathogenic evidence and the availability of independent objective markers to identify those at risk, already converted and beyond (Figure 1). While the motor features continue to be the primary criteria for identification of PD, the last few years has seen a surge for the development of objective and independent diagnostic and prognostic biological markers for PD, especially for the asymptomatic phases. A biomarker is defined as “a characteristic that is objectively measured and evaluated as an indicator of normal biological processes, pathogenic processes or pharmacologic responses to a therapeutic intervention” (58).

A growing number of markers have been proposed as effective screening tools for PD, including clinical, imaging, biochemical, and genetic (59). Different types of markers focus on specific features of PD, such as signs and symptoms, structural and functional integrity, accumulation and aggregation of abnormal proteins and other products of molecular processes as well as variations in the genetic make-up. Therefor, some biological markers are more applicable than others in certain phases of PD as they span the entire disease course, from the risk phase to clinical expression.

Although a multitude of biomarkers for PD have been proposed no biomarker can definitively predict PD onset. Some markers are more focused on the earliest phases of PD than others, but each could provide unique information regarding the presence and progression of PD. Crucially, individual biomarkers may lack sensitivity and specificity for accurate diagnosis and combinations of biomarkers implemented at the right time may be needed to achieve this. More importantly, the validation of individual and combinations of biomarkers is required for early diagnostic potential (59).

## The Way Ahead

We are making great strides in the efforts to understand the complexity of PD and the subsequent implications for the development of new diagnostic, prognostic and therapeutic methods, but many questions remain. We now know that the motor phenotype of PD is merely a milestone in a far more extended disease trajectory. Although at some point, most cases converge to increasing levels of movement difficulties and functional impairment in the course of the disease, the underlying cause, pathological pathways and molecular mechanisms might be considerably different, which needs to be reflected by future identification, stratification and therapeutic strategies.

Paradoxically, objective diagnostic tools are needed for intervention with new therapies when it matters most, but development of new therapies to effectively change the disease course requires new objective diagnostic tools. One intermediate way to deal with this paradox is to focus on the populations with an above average pre-disposition for developing PD, such as those with a genetic susceptibility or those with disorders like RBD or olfactory dysfunction that are known for a high risk of conversion to the PD phenotype. Prospective studies in these groups could subsequently inform most optimal therapeutic strategies aimed at modification and protection. In turn, these results can then inform new strategies in the treatment of sporadic forms of PD.

In the absence of a cure for PD, the Holy Grail seems the development of new therapies that impact the actual pathological processes. Although disease modification has successfully been shown in PD models, we are not sure if these treatments will ever work in humans. Regardless of whether it is possible, a lot of work can still be done to increase the effectiveness of current symptomatic therapies aimed at maintaining quality of life and wellness. Especially when we learn how to stratify cases more effectively and use this information to tailor symptomatic approaches to maximize impact on patients' quality of life and wellness. Fundamentally what is needed to move forward in our search for PD solutions is a better understanding of the natural progression of PD and the underlying pathological processes and mechanisms.

## Author Contributions

PP and GM contributed the conception and design of the manuscript. PP wrote the first draft of the manuscript. PS, KO'M, and GM wrote the sections of the manuscript. All authors contributed to the article and approved the submitted version.

## Conflict of Interest

The authors declare that the research was conducted in the absence of any commercial or financial relationships that could be construed as a potential conflict of interest.
